# Features of Uranium Recovery from Complex Aqueous Solutions Using Composite Sorbents Based on Se-Derivatives of Amidoximes

**DOI:** 10.3390/gels12010084

**Published:** 2026-01-18

**Authors:** Eduard A. Tokar’, Anna I. Matskevich, Konstantin V. Maslov, Veronika A. Prokudina, Alena N. Popova, Dmitry K. Patrushev

**Affiliations:** 1 Institute of Natural Sciences and Technosphere Safety, Sakhalin State University, Sakhalin Region, Yuzhno-Sakhalinsk 693000, Sakhalin Oblast, Russia; mysmatskevich@mail.ru (A.I.M.); veronikaprokudina2002@mail.ru (V.A.P.); alena.nikonovna@gmail.com (A.N.P.);; 2 Institute of High Technologies and Advanced Materials, Far Eastern Federal University, Vladivostok 690922, Russky Island, Primorsky Krai, Russia

**Keywords:** amidoxime, uranium, chitosan, seawater, sorption, radioactive waste

## Abstract

The article presents a comprehensive comparative performance evaluation and validation of composite adsorbents based on the Se-derivative of 4-amino-N′-hydroxy-1,2,5-oxadiazole-3-carboximidamide for U (VI) recovery from complex multicomponent aqueous media. Our results indicate the composite materials to be comparable to, and in some cases to surpass, existing adsorbents in recovery efficiency. Under static sorption conditions for trace U (VI) from real multicomponent solutions (tap, river, and sea water), the sorption efficiency reached 80–98%, while the distribution coefficients ranged from 10^4^ to 10^6^ cm^3^ g^−1^. The sorption-selectivity properties of the materials were evaluated in the presence of competing ions (EDTA and oxalate ions), which possess a high chelating capacity and a strong tendency to form complexes with uranium. The dependence of sorption efficiency on the concentration of these ions and the solution pH was investigated. The possibility of reusing the materials over multiple sorption-desorption cycles was assessed. An optimal regenerating eluent agent was identified (NaHCO_3_/NH_4_NO_3_), providing a desorption efficiency of >95% without degrading the material’s sorption properties over repeated cycles. Using a combination of physicochemical methods, including sorption techniques, the mechanism of uranium sorption and its dependence on the material structure were determined. The efficiency of uranium recovery from multicomponent natural waters was also investigated under dynamic conditions over repeated sorption-desorption cycles. The results demonstrate through comparative analysis that the developed composites exhibit a high sorption capacity and possess a high practical potential for the concentration and recovery of uranium from high-salinity solutions with complex composition.

## 1. Introduction

Rising global energy needs and the imperative to shift to a low-carbon economy lead to the growing interest in nuclear energy. Uranium, being the primary fuel for nuclear power, is central to this endeavor. However, traditional uranium ore mining is associated with significant environmental risks and economic costs. The quality of uranium deposits is also decreasing [1]. In this context, interest is growing towards the utilization of alternative sources of this strategic metal, including seawater, spent nuclear fuel solutions, mine and groundwater, and industrial effluents with complex salt compositions.

The primary obstacle to uranium extraction from such media is its exceptionally complex chemical environment, characterized by: ultralow uranium concentrations (less than 4 μg L^−1^ in river water, about 4 μg L^−1^ in seawater, and 5 μg L^−1^ in groundwater) along with high concentrations of competing ions (Na^+^, K^+^, Ca^2+^, Mg^2+^, Cl^−^, SO_4_^2−^, etc.), the presence of organic impurities, a wide pH range, and high salinity (up to 35 g L^−1^). Uranium’s high migration mobility, which depends on redox conditions, pH, and solution composition, is due to its specific physicochemical properties [2]. Conventional techniques, including ion exchange, liquid–liquid extraction [3,4], and precipitation [5], are often unsuitable for this application because of poor efficiency, high cost, and associated environmental risks.

Adsorption is one of the most promising approaches for uranium extraction, as it offers simplicity, cost-effectiveness, potential for sorbent regeneration, and low probability of generating secondary waste [6,7]. Among adsorbents with various functional groups capable of binding U (VI) ions, amidoxime groups (—C(NH_2_)=NOH) demonstrate the highest efficiency and selectivity towards the target radionuclide [7,8]. The amidoxime functional group, synthesized from the reaction of hydroxylamine or hydrazine with a nitrile group (–C≡N), exhibits high selectivity for uranium ions through a chelation mechanism [9].

However, the application of purely polymeric or fibrous amidoximated sorbents is often limited by their low mechanical strength and insufficient sorption kinetics. A key strategy to overcome these limitations is the creation of composite adsorbents, which synergistically combine the advantages of a support matrix and amidoxime groups. Such composites demonstrate high equilibrium adsorption capacity for uranium, which can reach 300–500 mg g^−1^ [10] or more in model solutions. However, the choice of matrix is critical for determining the mechanical and kinetic properties of the final product.

The selectivity of amidoxime-based composites can be enhanced through several strategies. One approach involves creating a hydrophobic microenvironment around the amidoxime groups, which restricts the access of hydrated competing cations (Mg^2+^, Ca^2+^). Another strategy is the introduction of additional specific ligands, such as phosphonate or carboxyl groups [11], which creates a synergistic effect by combining different uranium-binding mechanisms.

A wide range of inorganic materials are commonly employed as substrates, including: magnetic nanoparticles based on iron oxides (Fe_3_O_4_) [12,13], mesoporous silica [14,15], metal–organic frameworks (MOFs) [16,17,18], carbon materials (graphene [19], carbon nanotubes, activated carbon [20]), and natural minerals (zeolites, montmorillonite [21,22]). The use of such materials as matrices is motivated by their high specific surface area (500–700 m^2^ g^−1^ [20,23]), and thermal and mechanical stability. Additionally, magnetic (such as magnetic nanoparticles-based materials [12,13]) adsorbents can be separated via an external magnetic field, eliminating the need for labor-intensive post-sorption techniques like filtration or centrifugation. However, most composites with an inorganic matrix do not exhibit high sorption capacity (typically up to 200–300 mg g^−1^), and their operational capabilities are strictly limited to an acidic environment (pH 2–6). At higher pH, the sorption process is accompanied by reduced selectivity towards uranium due to the strong competing effect of accompanying ions and partial dissolution of the sorbent material.

Thus, the application of inorganic matrix-based composites is significantly limited. Their most substantial drawback is relatively low selectivity in high-salinity alkaline environments, such as seawater—a complex, multicomponent system where the concentration of competing ions (primarily Ca^2+^ and Mg^2+^) exceeds that of uranium by four to five orders of magnitude. The sorption kinetics are of critical importance for uranium extraction from seawater, a consequence of the low concentration of the target ion. The kinetic parameters of sorbents can be optimized by using organic and organometallic matrices. The use of amidoxime-functionalized materials for this purpose is justified by their high affinity for various ionic forms of uranium, which is several orders of magnitude greater than that for the main competing ions.

Porous polymer beads (e.g., based on styrene-divinylbenzene [23]), polymer fibers (polypropylene [22], polyacrylonitrile (PAN)), or gels (polyvinyl alcohol [22]) are commonly used as substrates. Polymeric matrices provide flexibility, ease of modification, and a high density of functional groups. For instance, the authors [22] fabricated super-crosslinked hydrogel fibers based on polyamidoxime (PAO) and polyvinyl alcohol (PVA) hydrogel via electrospinning and vapor-phase crosslinking (c-PAO/PVA NF). This synthesis technique enhanced the material’s hydrophilicity by incorporating PVA and extending the PAO molecular chains, which increased the density of sorption sites. Evaluation of the pH dependence revealed that uranium sorption efficiency rose from pH 4 to pH 6, peaking at 364 mg g^−1^. However, dynamic sorption experiments using simulated seawater demonstrated a decline in the total dynamic exchange capacity to 134 mg g^−1^ after 30 h, suggesting a loss of selectivity toward the radionuclide, attributable to partial adsorbent dispersion.

Natural matrices, including polymers, chitosan, cellulose, and their modifications, are also widely used for uranium sorption. The presence of inherent ion-exchange groups (-OH and -NH_2_) in these matrices not only allows for the firm anchoring of modifier functional groups during composite synthesis but also contributes to enhanced sorption capacity by synergetic effects. According to reference [24], to increase the number of active sites, chitosan was functionalized via two routes. The first involved direct amidoximation of hydroxyl groups, and the second involved the immobilization of macromolecular ligands (polyethylene polyamine and polydopamine). This combined approach significantly increased the material’s sorption capacity, reaching 470 mg of uranium per gram of sorbent.

Despite the wide variety of synthesized materials with high sorption-kinetic characteristics for uranium, the challenge of its efficient extraction from highly mineralized aqueous media remains unresolved. A clear illustration of this problem, as discussed earlier, is their performance in seawater or its simulants: under these conditions, the selectivity of most adsorbents decreases sharply, leading to a 10–100-fold drop in static capacity (2–20 mg g^−1^) [25,26,27,28,29,30,31].

We have previously demonstrated that Se- and S-derivatives of 4-amino-N′-hydroxy-1,2,5-oxadiazole-3-carboximidamide are highly effective sorption-active components for uranium binding in liquid media [32]. Moreover, we have demonstrated that the performance of sorption can be significantly improved by incorporating the Se-derivative into composite materials. Immobilizing the Se-derivative onto a mineral matrix (silica gel) [33] or covalently grafting it onto chitosan [34] leads to a 2–10-fold improvement in sorption kinetics relative to the pristine, non-composite material, owing to their developed surface morphology, which provides greater accessibility of the sorption centers and improves overall sorption capacity.

We established that in model single-component solutions, these materials can remove uranium over a wide pH range. At an initial U (VI) concentration of 20–30 mg L^−1^, the maximum in sorption capacity was achieved in the pH range of 4–8, where the sorption efficiency (S, %) exceeded 95%. The observed decrease in efficiency at lower (pH < 4) or higher (pH > 8) values is attributed to the competitive protonation of functional groups and the partial dissolution of the sorption-active component of the material, respectively.

For the chitosan-based composite, the highest U (VI) distribution coefficients (Kd, mL g^−1^) were recorded (Kd = 10^5^–10^6^ mL g^−1^). This is explained by the high ability of the radionuclide to be sorbed not only on the surface but throughout the entire volume of the sorbent grain due to the high ionic permeability of the polymer matrix.

The previously obtained results allow us to conclude that composite materials based on 4-amino-N′-hydroxy-1,2,5-oxadiazole-3-carboximidamide combined with organic or inorganic matrices possess promising performance characteristics for practical application in uranium removal from natural and anthropogenic aqueous media.

This study is focused on a comparative performance evaluation and validation of the previously developed Se/Si-50 and Chit-1/1 composites under conditions close to real applications. This stage of the research is focused on determining the sorption-selectivity properties of the composite materials for uranium in complex-composition, high-salinity simulant solutions, along with elucidating the specific features of the uranium binding mechanisms, and establishing the optimal operational parameters for radionuclide removal under dynamic conditions.

## 2. Results and Discussion

Based on the results of our previous work, which demonstrated that the maximum in sorption efficiency is achieved at a 1:1 molar or mass ratio of the support matrix to the Se-derivatives of amidoxime (for chitosan-Se-am (Chit-1/1) or SiO_2_-Se-am (Se/Si-50) composites, respectively), the composite materials prepared at this specific ratio were selected for the experiments in this study. To evaluate the effect of compositing, the non-composite adsorbent (Se-am) was used as a reference.

### 2.1. Sorption from Model and Real Solutions

**Figure 1:**
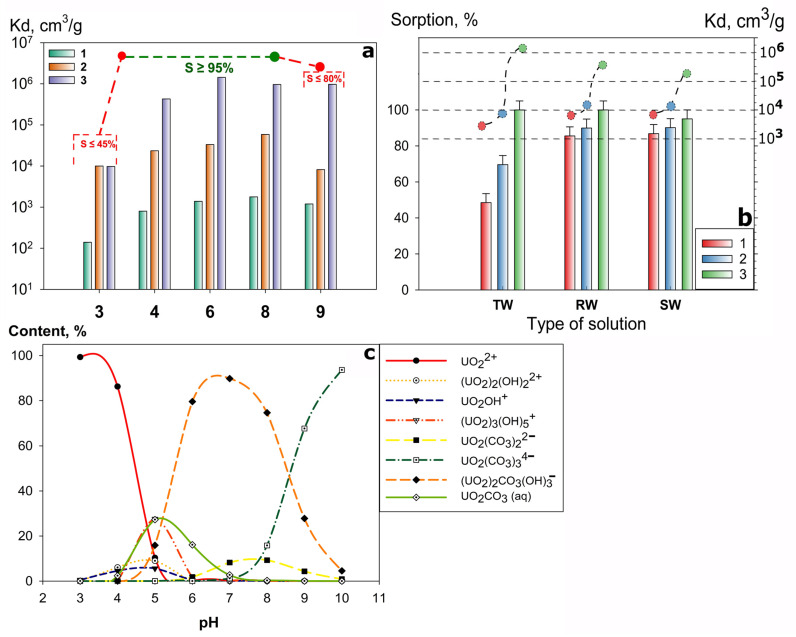
Uranium sorption efficiency (**a**) as a function of pH in a single-component system and (**b**) as a function of solution composition in multicomponent systems. 1—Se-am, 2—Se/Si-50, 3—Chit-1/1, at a V/m ratio of 1000 cm^3^ g^−1^ and uranium concentration 1 ng L^−1^. (**c**) Calculated distribution of uranium ionic species as a pH function (Visual MINTEQ). The dotted lines in (**a**) show the integral change in the sorption efficiency (S, %) with a change in the pH of the solution; (**b**) shows the integral change in the K_d_ value between the sorbents.

The initial phase of the study focused on the sorption of U (VI) from a model monocomponent solution using the investigated sorbents. The results (Figure 1a) indicate that all materials effectively recover the radionuclide across a wide pH range, with the maximum sorption capacity (S > 95%) being achieved at pH 4–8. The decrease in sorption efficiency beyond this range is caused by the competing protonation reaction in acidic media and the partial dissolution of the materials under alkaline conditions.

The high Kd values for U (VI) for the Chit-1/1 composite (Kd = 10^5^–10^6^ mL g^−1^), which significantly exceed those of other sorbents, are due to the high ion permeability of its matrix. This property enables the sorption of the radionuclide not only on the surface but throughout the entire volume of the sorbent grain. During the U (VI) recovery from more chemically complex, multicomponent real solutions—tap water (TW), river water (RW), and seawater (SW)—the sorption-selectivity properties of the adsorbents decreased (Figure 1b). The sorption efficiency and Kd values decreased in the order TW ≥ RW ≥ SW. This effect is associated with the increasing influence of competing ions and the formation of polynuclear uranium complexes (Figure 1c) [35], resulting from the higher salinity of the solutions. The highest K_d_ values (10^5^–10^6^ cm^3^/g) were achieved for all solutions using Chit-1/1, which is attributed to the presence of additional sorption-active centers (-NH_2_ and -COOH).

All sorbents exhibited a decrease in U (VI) sorption efficiency from TW (Figure 1b). This is probably associated with high content of iron ions and the presence of complexing agents from the water treatment process, which act as strong competing agents. However, no significant drop in sorption efficiency was observed for the SW solution, indicating the materials’ enhanced selectivity toward uranium.

### 2.2. Effect of Complexing Agents (EDTA, Oxalate) on Uranium Sorption

The efficiency of uranium removal is critically dependent on the ionic composition of the solution. This dependence is directly related to uranium’s propensity to form a variety of complex species. In our previous works [33,34], we established that the inhibitory effect of common competing ions increases in the order: Cl^−^ ≈ NO_3_^−^ ≤ PO_4_^3−^ ˂ SO_4_^2−^ ˂ HCO_3_^−^ ≈ CO_3_^2−^. However, because of intensive industrial activity, natural and technogenic liquid media may contain other, more potent complexing agents. For instance, EDTA and oxalate ions exhibit high metal-chelating capacity, which is commonly used in various technological processes, including uranium mining and processing. Despite their similar metal-binding ability, their environmental roles and ultimate effects differ. EDTA typically forms stable, water-soluble complexes, which mobilize metals and can facilitate their spread in the environment, posing serious ecological risks. In contrast, oxalate often leads to the precipitation of metals as insoluble compounds, ensuring their immobilization [36]. In this context, a relevant and timely problem is evaluating the impact of these chelating agents on the uranium sorption parameters for our sorbents.

**Figure 2:**
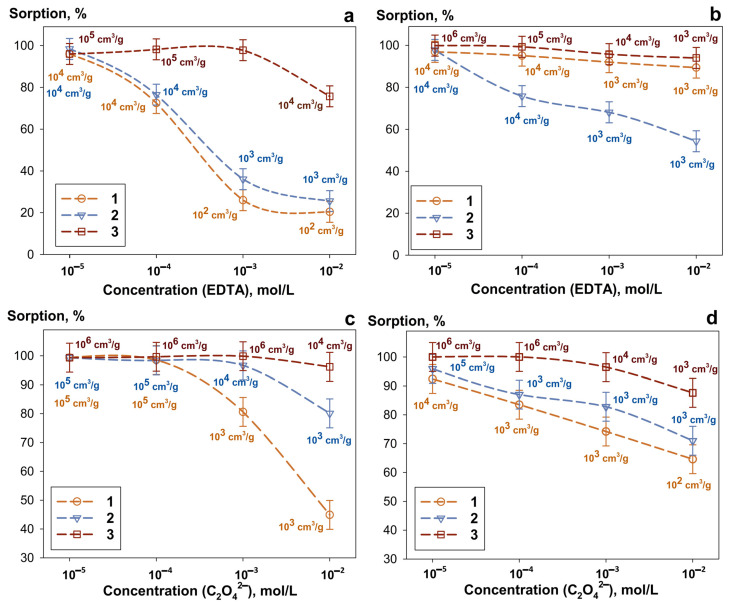
Dependence of uranium sorption efficiency on complexone concentration. (**a**,**b**) EDTA and (**c**,**d**) C_2_O_4_^2−^ in pH 6 (**a**,**c**) or pH 8 (**b**,**d**) solutions. 1—Se-am, 2—Se/Si-50, 3—Chit-1\1 (V/m ratio—1000 mL g^−1^).

Figure 2 demonstrates the uranium sorption efficiency as a function of the type and concentration of complexing agents for solutions at pH 6–8. In the presence of EDTA, a decrease (by an order of magnitude) in the K_d_ values was observed with increasing solution pH. This trend is attributed to the enhanced deprotonation reaction of the complexing agent and the low degree of uranium hydrolysis. This effect was amplified with increasing EDTA concentration, which is associated with the formation of bulky polynuclear EDTA—U complexes. These complexes are energetically and spatially hindered from forming chemical bonds with the amidoxime functional group. Similar results were obtained in the presence of oxalate ions. The sorption selectivity among the adsorbents decreases in the order: Chit-1/1 ˃˃ Se\Si-50 ≥ Se-am. The K_d_ values remain in the range of 10^3^–10^6^ mL g^−1^, and the sorption efficiency does not fall below 80% at a complexing agent concentration of 0.01 mol L^−1^.

### 2.3. Study of Sorption Kinetic Parameters

In dynamic sorption processes, the sorption rate is a critical parameter limited by uranium mass transfer to the sorbent’s active sites. This characteristic is directly determined by the material’s physicochemical properties. To determine the mass transfer mechanism, uranium sorption kinetics were studied under both continuous and interrupted conditions. The interruption protocol involved removing the sorbent from the model solution after 32 min and reintroducing it after 48 h.

**Figure 3:**
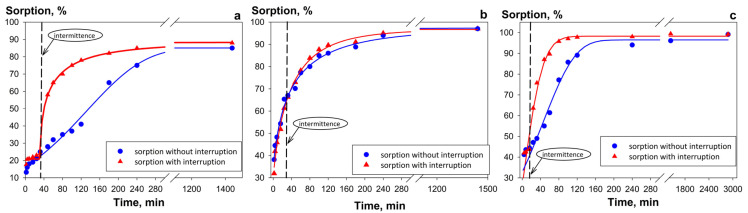
Sorption kinetics of U (VI) from model solution (pH 8, 0.01M NaHCO_3_) with (**a**) Se-am, (**b**) Se/Si-50, (**c**) Chit-1\1. Experiments performed in continuous and interrupted regimes (V/m ratio—2000 mL g^−1^).

Results for the initial sorbent Se-am (Figure 3a) indicate that equilibrium was reached after 160 min in the interrupted experiment, compared to 280 min in the continuous mode. This difference indicates that the sorption kinetics are limited by intraparticle diffusion. The interruption promoted the redistribution of uranium ions from the sorbent surface into its grain volume, thereby accelerating the attainment of equilibrium. This observation confirms the presence of ion permeability in the sorption-active component.

In contrast to the initial Se-am adsorbent, the porous composite Se/Si-50 reached maximum sorption efficiency within 240 min in both cases, regardless of experiment interruption (Figure 3b). This is attributed to the specific structural features of the sorbents. During synthesis, a film of the active component several tens of nanometers thick forms on the surface of the SiO_2_ matrix. The small volume of the Se-am grains reduces the contribution of slow gel diffusion to the overall process kinetics. Consequently, the process is limited by external diffusion mass transfer at the “model solution—sorbent surface” phase boundary, which depends primarily on the specific surface area and grain size of the Se/Si-50 sorbent.

The highest sorption rate was achieved with Chit-1/1 composite (Figure 3c). The time required to achieve the maximum sorption efficiency (>95%) for this material is 2–3 times shorter, amounting to 80 and 120 min for the interrupted and uninterrupted experiment modes, respectively (Table 1). The high ionic permeability of chitosan and the uniform distribution of the active component (Se-am) throughout the grain volume allow for reduced potential limitations posed by internal diffusion mass transfer, thereby accelerating the sorption kinetics. An increase in specific surface area and pore volume significantly enhances adsorption kinetics. This is evidenced by a reduction in both the equilibrium time and the half-exchange time, along with an increase in diffusion coefficient values. The superior kinetic and sorption parameters of the Chit-1/1 confirm that a highly porous structure with large-sized pores is the key to developing efficient sorbents with high target ion recovery rates.

### 2.4. Thermodynamic Parameters of Sorption

**Figure 4:**
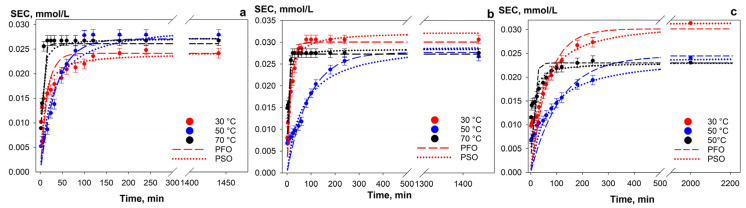
Sorption kinetics of U (VI) from a model solution (pH 8, 0.01 M NaHCO_3_) at V/m = 500 mL g^−1^ and different temperatures with (**a**) Se-am, (**b**) Se/Si-50, (**c**) Chit-1/1 (V/m ratio—2000 mL g^−1^).

Figure 4 demonstrates the sorption kinetics obtained under static conditions at different solution temperatures. The kinetic data were used to calculate the rate constants for the pseudo-first-order and pseudo-second-order models, along with the experimental SEC values (Table 2). The frequency of aliquot sampling after 300 min was reduced due to the absence of significant changes in concentration.

The sorption kinetics were best described by the pseudo-second-order model, which indicates that the uranium sorption process onto the Se-am active component is complex, being influenced by both intraparticle mass transfer and the exchange processes at the sorbent’s functional groups. This is particularly evident for the Chit-1/1 sample (Figure 4c). The pseudo-second-order rate constant increases in the order: Se-am < Se/Si-50 < Chit-1/1. This trend suggests an enhancement in the exchange process rate through the presence of a porous structure or the higher ion-exchange capacity of the matrix.

Composite materials also demonstrate a higher SEC value, indicating greater accessibility of the sorption sites. The decrease in sorption capacity with increasing temperature directly confirms the exothermic nature of the process. For the Chit-1/1 sample, an additional decrease in SEC values with increasing temperature is attributed to uranium desorption, which is caused by partial degradation of the polymer matrix. The destruction was confirmed by the formation of a specific polymer gel above the sorbent after settling.

### 2.5. Sorbents Stability in Sorption–Desorption Cycles

An important characteristic of a sorbent is its potential for multiple uses in sorption-desorption cycles. However, selecting a regenerant solution is crucial. It must provide sufficient desorption efficiency without causing material degradation or loss of its original properties.

**Figure 5:**
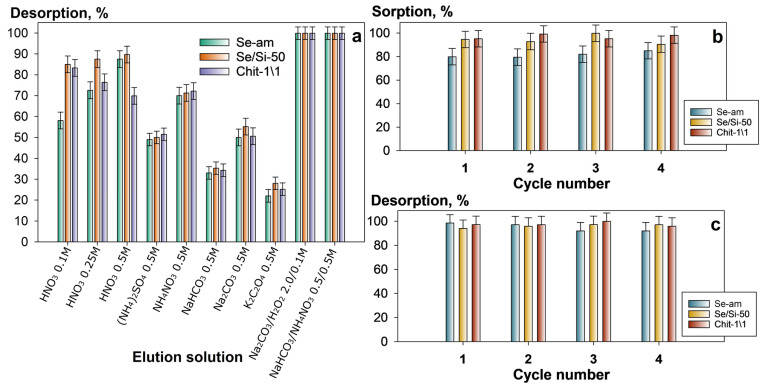
Uranium desorption efficiency for different eluent solutions (**a**); uranium sorption and desorption efficiency over repeated cycles of sorption from a 0.01 M NaHCO_3_ model solution and desorption using a 0.5 M NaHCO_3_ and 0.5 M NH_4_NO_3_ solution (**b**,**c**), V/m ratio—2000 mL g^−1^).

We investigated the possibility of regenerating the sorbent materials using solutions of different compositions (Figure 5a). The type and concentration of the eluent were selected based on literature data from related studies [37,38] and the chemical properties of uranium and the analyzed adsorbents. The uranium desorption efficiency decreased in the order: NaHCO_3_/NH_4_NO_3_ ≈ Na_2_CO_3_/H_2_O_2_ ˂ HNO_3_ ˂ NH_4_NO_3_ ˂ Na_2_CO_3_ ≤ (NH_4_)_2_SO_4_ ˂ NaHCO_3_ ˂ K_2_C_2_O_4_.

Varying the concentration of nitric acid (commonly used agent for adsorbent regeneration) from 0.1 to 0.5 mol L^−1^ did not lead to significant changes in uranium desorption efficiency, which did not exceed the 90%. Despite the high desorption efficiency of the Na_2_CO_3_/H_2_O_2_ solution, this option is unsuitable because of the partial dissolution of the adsorbent material caused by the oxidation of the amidoxime by H_2_O_2_. In our opinion, the most suitable desorption agent is the solution containing 0.5 mol L^−1^ NaHCO_3_ and 0.5 mol L^−1^ NH_4_NO_3_. Further experiments utilized this solution. The high desorption efficiency achieved with the solution is probably due to the highly competitive capacity of NH_4_^+^ ions, which displace uranyl ions. These ions subsequently form stable polynuclear carbonate complexes, such as UO_2_(CO_3_)_3_]^4−^, [UO_2_(CO_3_)_4_]^3−^, and [UO_2_(CO_3_)_2_(H2O)_2_]^2−^, thereby preventing the re-sorption of uranium from the eluent solution. In all cases, the maximum desorption efficiency was reached within 60–120 min.

Figure 5b,c demonstrate the results of testing the adsorbents in repeated sorption-desorption cycles. A model solution containing 30 mg L^−1^ U (VI) in 0.01 mol L^−1^ NaHCO_3_ at pH 8 was used for the sorption phases. The sorption efficiency remained stable throughout all cycles, with values of 80%, 90%, and 98% for Se-am, Se/Si-50, and Chit-1/1, respectively. No dissolution of the adsorbents was observed during the described experiments, as confirmed by the absence of Se in the filtrates after the sorption-desorption cycles. Moreover, the gravimetric analysis results confirmed the absence of any degradation or dissolution of the sorbent materials: in parallel experiments, the sorbent was dried and weighed after the regeneration stage. No significant changes in the sorbent mass were recorded.

### 2.6. Study of Uranium Sorption Under Dynamic Conditions

**Figure 6:**
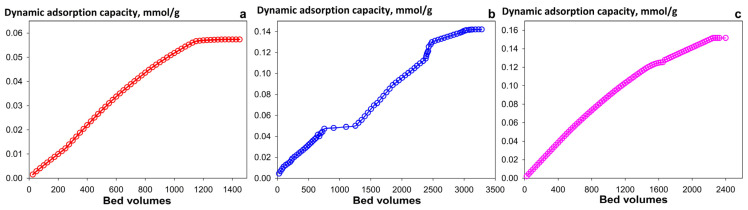
Accumulation curves of U (VI) under dynamic conditions: (**a**) Se-am, (**b**) Se/Si-50, (**c**) Chit-1/1. Conditions: 0.01 M NaNO_3_ solution, pH 8, particle size 0.1–0.2 mm, flow rate 5 BV/h.

For practical applications, such as treating liquid waste or extracting uranium from liquid media for mining purposes, the flow-through method is more optimal. The key advantage of this approach is that it ensures process continuity. In this regard, we have studied characteristics of the sorbents under dynamic sorption conditions. Figure 6 demonstrates the integral curves for U (VI) sorption from a 0.01 mol L^−1^ NaNO_3_ solution at pH 8 under dynamic conditions. The results indicate that the composite materials exhibit a more than twofold higher total dynamic exchange capacity (TDEC) than the pristine Se-am adsorbent, consistent with trends observed in static sorption experiments. The TDEC decreases in the order: Chit-1/1 > Se/Si-50 > Se-am. The change in the uranium adsorption curve for Se/Si-50 (Figure 6b) corresponds to a shift from an external to an intra-particle diffusion-limited rate regime. Initially, rapid sorption occurs on the most accessible surface sorption sites. The process then slows as it becomes limited by the diffusion of uranium ions into the active component nanofilm coated on the SiO_2_ particles. The diffusion is further retarded under conditions of high ionic strength. Consequently, the inflection point on the curve reflects the transition from fast surface sorption to a rate-limiting step governed by intra-particle diffusion and competing complexation. For the Chit-1/1 composite (Figure 6c), this effect is less pronounced due to the high ionic permeability of the chitosan matrix and the uniform distribution of active sites throughout the particle volume. These features ensure faster kinetics and result in a smoother adsorption curve.

**Figure 7:**
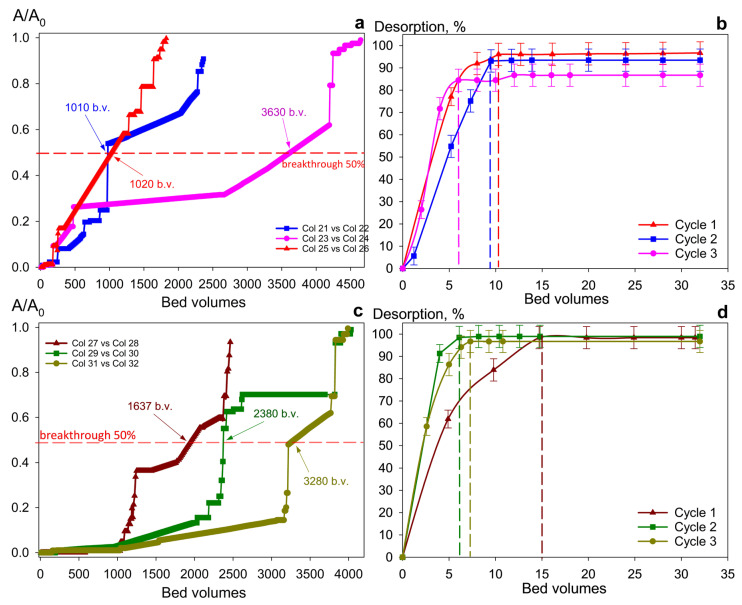
U (VI) recovery from river water in repeated cycles. (**a**,**c**) sorption and (**b**,**d**) desorption under dynamic conditions with (**a**,**b**) Se/Si-50 and (**c**,**d**) Chit-1/1. Conditions: particle size 0.1–0.2 mm, flow rate 10 BV/h.

**Figure 8:**
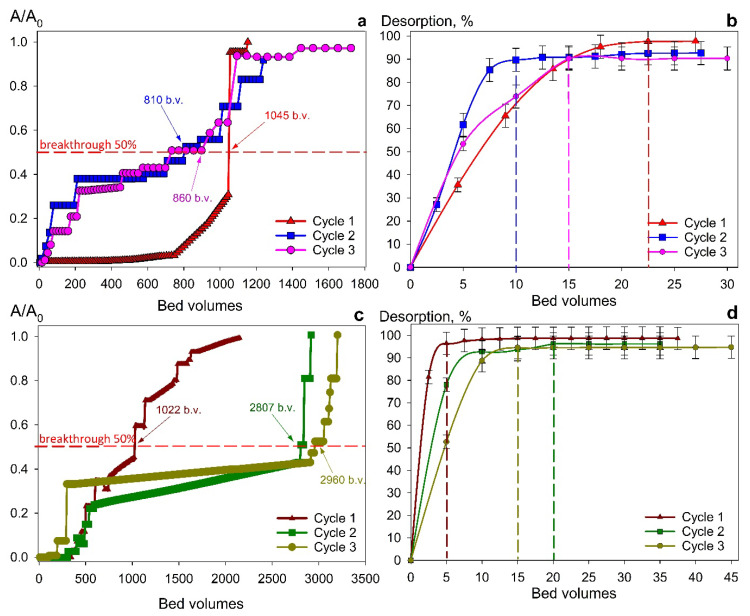
U (VI) recovery from seawater in repeated cycles. (**a**,**c**) sorption and (**b**,**d**) desorption under dynamic conditions with (**a**,**b**) Se/Si-50 and (**c**,**d**) Chit-1/1. Conditions: particle size 0.1–0.2 mm, flow rate 10 BV/h.

The Se-am material was excluded from further dynamic studies because of its lower sorption capacity and operational difficulties caused by fine particle size, which creates high hydrodynamic resistance. Consequently, all subsequent experiments were performed using only Se/Si-50 and Chit-1/1 composites. Figure 7 and Figure 8 demonstrate the uranium accumulation curves from repeated sorption-desorption cycles with real RW and SW solutions, respectively. Both adsorbents show increasing sorption capacity from the first to the third cycle, probably due to the gradual removal of unreacted synthesis products and the conversion of sorption sites to a uniform ionic form. These effects also reflect on desorption process characteristics: each subsequent cycle requires less eluent volume for complete uranium recovery.

The materials are characterized by high selectivity for ionic uranium species in solutions with a complex salt composition (Table 3). In river water treatment, the effective filtration capacity (defined as the point of 50% uranium breakthrough) reached 3280 bed volumes for Chit-1/1 and 3630 for Se/Si-50 (Figure 7a,c).

Switching from river water (Figure 7a,c) to seawater (Figure 8a,c), a threefold decrease in dynamic exchange capacity (to 1040 bed volumes) is observed for the Se/Si-50 sorbent. This is related to the higher salinity background and the resulting intensification of reactions that compete with uranium binding reactions. In contrast, this effect is minimal for the Chit-1/1 sorbent. We assume that this effect is caused by the chitosan matrix itself, which exhibits a strong and selective complexation behavior toward uranium. The desorption efficiency exceeded 90% in all sorption-desorption cycles. However, compared to river water, the eluent volume required for complete sorbent regeneration after seawater treatment nearly doubled (Figure 8b,d). This is probably due to some features of the formation of salt deposits on the adsorbent grain surface through the generation of polynuclear uranium complexes such as [Se/Si-50] —U—CO_3_—U—X (where X = X = CO_3_^2−^, SO_4_^2−^, Cl^−^, etc.). Significantly, during the dynamic tests, no selenium leaching into the filtrate was observed, which indicates the chemical stability of the material and suggests its potential for safe use.

Table 3 summarizes literature data on uranium sorption under dynamic conditions for various sorbent types. Notably, among the wide range of sorbents reported, studies on radionuclide sorption in dynamic mode using natural aqueous media or their imitation are relatively scarce. Furthermore, a direct quantitative comparison or ranking of sorbents based on these literature data is often limited due to significant variations in experimental protocols across different studies (e.g., initial U (VI) concentration, flow rate, particle size, solution composition).

This comparison, however, highlights the competitive advantages of the adsorbents developed in this work. They demonstrate not only higher values of total dynamic capacity and effective filtration cycles but also high stability over multiple sorption-desorption cycles without loss of efficiency, which are critical parameters for practical application. But we recognize that for a more comprehensive and accurate assessment of the sorption-selective properties of the adsorbents toward U(IV) under real-world conditions, our subsequent research should focus on uranium sorption at concentrations approximating those found in global seawater (~3.3 μg L^−1^).

### 2.7. Study of the Mechanism of Uranium Binding

In the next research stage, the mechanisms of uranium binding were studied to inform sorbent optimization. Table 4 shows the results of calculating the interplane distance and the size of the coherent scattering region in the obtained adsorbents before and after sorption of U (VI) from a monocomponent solution with pH 6.

Before analyzing the results, it is important to address the key assumptions of the computational model used to determine the structural parameters. In polymer systems, values obtained by the Scherrer method reflect not the true crystallite size, but the average size of coherently scattering domains perpendicular to the diffraction planes. This is because a “crystalline region” in a polymer typically represents a lamella or a domain within a complex supramolecular structure (e.g., a spherulite), rather than an isolated grain. These parameters are not absolute material characteristics but serve as a valuable comparative tool for tracking changes during the sorption process. It is important to note that for amorphous polymers and composites, the shifts in the interplanar spacing (d) and CSR size should not be interpreted as direct evidence of sorbent recrystallization. Instead, it is more accurate to regard them as qualitative indicators of the redistribution of local density and the induction of order within nanoscale regions of the material under the influence of the sorbate. In this study, the described approach was used specifically to compare the “adsorbate-adsorbent” interaction mechanisms for the analyzed materials.

It has been established that uranium adsorption by the Se/Si-50 composite leads to a decrease in the interplanar spacing (d) and an increase in the size of the coherent scattering region (CSR). This can be considered evidence of sorption-induced ordering and strengthening of the structure [45,46]. The adsorbate acts as an external factor that relieves internal stresses and initiates recrystallization (or repolymerization). This process results in the formation of a denser matrix (lamellae) and may also contribute to the collapse of pores on the surface of the sorption-active component.

The calculation of kinetic parameters for Se-am and Chit-1/1 indicated that radionuclide binding is limited by intraparticle diffusion (Table 4). This is confirmed by characteristic changes in the molecular structure’s physical parameters after uranium sorption. For these materials, an increase in the interplanar spacing and a decrease in the CSR size were observed, indicating a non-uniform impact of the adsorbate on the molecular structure. The incorporation of uranium promotes material swelling, which creates mechanical stress at the boundaries of the ordered domains. As adjacent lamellae or blocks move apart, they can shift relative to each other, losing coherency. The disruption of long-range order is manifested by the “fragmentation” of large coherent scattering domains into several smaller, yet still ordered, fragments. Thus, the adsorbate does not merely passively fill voids but actively rearranges the material’s supramolecular organization. This leads to its partial destruction, namely, the “loosening” of the lattice, accompanied by the fragmentation of large ordered domains. The significant reduction in CSR size in Chit-1/1 indicates active filling of the pore space, which probably functions as a molecular sieve.

Comparative FT-IR spectra of the Se/Si-50 and Chit-1/1 materials before and after uranium sorption are presented in Figure 4. The observed shifts in the peak positions in the spectra following U (VI) sorption confirm the binding of the radionuclide to the functional groups of the sorbents.

**Figure 9:**
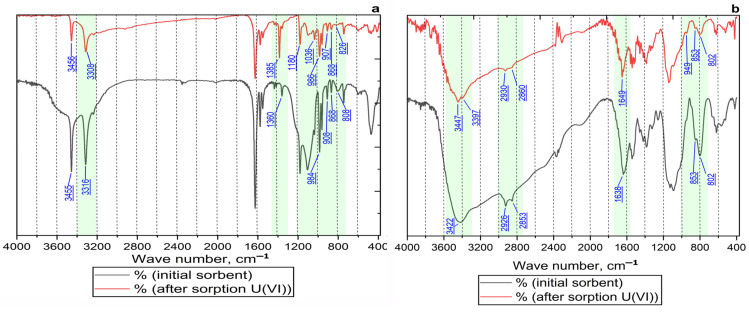
FT-IR spectra for sorbents before and after uranium sorption: (**a**) Se/Si-50, (**b**) Chit-1/1.

In the spectrum of the Se/Si-50 composite (Figure 9a), a shift in the absorption bands to lower wavenumbers was observed: the bands corresponding to the stretching vibrations of –OH and –NH_2_ groups shift from ~3455 cm^−1^ to ~3465 cm^−1^ and from ~3308 cm^−1^ to ~3316 cm^−1^, respectively. This indicates the involvement of hydroxyl (—OH) and amino (-NH_2_) groups in uranium ion binding [47]. The observed low-frequency shift in the O-H band provides direct evidence of chemisorption. This shift results from the weakening of the O—H bond through its coordination with the UO_2_^2+^ cation via the oxygen atom. Additionally, several spectral changes were observed after sorption. In the 1036–1180 cm^−1^ region, the intensity decreased for bands assigned to Si—O—Si (~1036 cm^−1^) and Si—O—C/C—O (~1180 cm^−1^) stretching vibrations. Additionally, the band for O-H/C-H deformation vibrations shifted from 1360 cm^−1^ to 1385 cm^−1^. These changes suggest the involvement of the silicate framework’s oxygen bridges and ester/alcoholic (C—O) groups in the interaction with uranyl ions, leading to a redistribution of electron density and changes in the force constants of the bonds.

The emergence of an intense band at ~986 cm^−1^ unequivocally identifies the symmetric stretching vibration of the O=U=O bond, confirming the presence of uranium on the surface in the form of the linear UO_2_^2+^ cation without a change in its oxidation state or the formation of other oxide species. The appearance of new bands at ~907 cm^−1^, ~868 cm^−1^, and ~826 cm^−1^, characteristic of the U—O-ligand stretching vibrations [48,49], provides further confirmation of coordination. The formation of strong U-O-ligand and U—NH_2_—ligand coordination bonds on the surface of the porous composite indicates an inner-sphere complexation mechanism, which explains the material’s high efficiency for uranium recovery from solutions.

For the Chit-1/1 sorbent (Figure 9b), a shift and decrease in intensity are observed for the bands at ∼2926 cm^−1^ and ∼2853 cm^−1^, characteristic of C—H stretching vibrations in the chitosan backbone. This indicates a change in the local environment of the polymer’s hydrocarbon skeleton, probably due to conformational rearrangements of the chitosan macromolecule induced by complex formation with uranium. This hypothesis is supported by the previous conclusion that the sorption process is limited by an intraparticle diffusion mechanism, facilitated by the high ionic permeability of the chitosan matrix.

The band at 1638 cm^−1^, corresponding to the —NH_2_ bending vibration (from both chitosan and the amidoxime component) and C=N stretching vibrations (imine or azomethine group) in the furan derivative, shifts to 1649 cm^−1^. This indicates the active participation of nitrogen atoms from both the polymer matrix and the grafted sorption-active component in uranium coordination.

The emergence of peaks in the 950–800 cm^−1^ region, along with the changes described above, confirms uranium coordination with the material’s functional groups: amino (—NH_2_) and hydroxyl groups of chitosan and amidoxime, as well as imine (C=N) and oximino (N–O) groups of the Se-amidoxime derivative. This results in crosslinking of the chitosan polymer chains via metal ions, which may induce conformational changes in the macromolecule and densification of the composite’s structure (Table 3).

## 3. Conclusions

This comparative validation study demonstrated that composite selenium-containing sorbents, specifically materials based on Se-derivatives of 4-amino-N′-hydroxy-1,2,5-oxadiazole-3-carboximidamide grafted onto SiO_2_ (Se/Si-50) or chitosan (Chit-1/1) matrices, exhibit high efficiency of U (VI) removal from liquid media with complex salt compositions, including real tap, river, and sea water. The uranium distribution coefficients in such multicomponent media reached 10^4^–10^6^ cm^3^ g^−1^.

Our results also demonstrated the analyzed materials to be comparable to several known adsorbents in uranium removal efficiency and to surpass them in several key parameters. Their sorption selectivity was shown to persist across concentration scales, from high to trace (µg L^−1^) levels. For instance, under static conditions, the uranium removal efficiency exceeded 98% across the pH range of 6–8, even at trace concentrations.

All tested materials maintained stable sorption performance across different uranium-containing solution compositions. The Chit-1/1 sorbent exhibited the highest uranium selectivity under high ionic strength conditions, including real seawater.

Upon introduction of strong complexing agents (EDTA, oxalate ions, 0.01M) into model solutions, the ranking of sorbents by efficiency was preserved: Chit-1/1 >> SiO_2_-50 > Se-am. Under these conditions, the distribution coefficient values ranged from 10^3^ to 10^6^ mL g^−1^, and the sorption efficiency remained above 80%.

For the polymeric sorbent Se-am, the sorption rate is limited by external diffusion. In contrast, for the porous materials Se/Si-50 and Chit-1/1, the rate-determining step is intraparticle diffusion. The enhanced sorption kinetics of the Chit-1/1 sorbent result from a synergistic effect that combines the high ionic permeability of the matrix with a uniform volumetric distribution of active sorption sites.

An optimal condition for sorbent regeneration was selected. It was demonstrated that an eluting solution of 0.5M NaHCO_3_/0.5M NH_4_NO_3_ provides a desorption efficiency exceeding 95% in all sorption-desorption cycles.

The total dynamic exchange capacity (TDEC) was 0.16, 0.06, and 0.014 mmol g^−1^ for Chit-1/1, Se/Si-50, and Se-am, respectively. The effective filter cycle capacity (until a uranium breakthrough of ≥50%) for river and seawater reached 3280/1040 and 3630/2980 b. v. for Chit-1/1 and Se/Si-50, respectively.

The results indicate the promising potential of the described sorbents for uranium recovery from liquid media, both for the remediation of radionuclide contamination and for its concentration as a valuable resource.

## 4. Materials and Methods

Especially pure grade selenium dioxide (SeO_2_), methylene chloride (CH_2_Cl_2_), ethanol (C_2_H_6_O), and acetic acid (CH_3_COOH) (Nevareaktiv LLC, St. Petersburg, Russia) were used to synthesize the analyzed adsorbents. Initial chitosan (Nevareaktiv LLC, St. Petersburg, Russia) had an acetylation degree of 0.25 and a viscosity-average molecular weight of 250 kDa. Model metal-ion-containing solutions were prepared from chemically pure salts (Nevareaktiv LLC, St. Petersburg, Russia) with no further purification. Sorption experiments at macroconcentration (20–30 mg L^−1^) levels of uranium were conducted using ^238^U (VI), prepared from high-purity uranyl nitrate (UO_2_(NO_3_)_2_), supplied by GRANKHIM LLC, St. Petersburg, Russia. For sorption experiments from trace uranium solutions (1 ng L^−1^), a mixture of uranium isotopes (^232^U and ^233^U) was used. For simplicity, the term “uranium” will be used throughout this article to refer to U (VI) and its possible chemical forms.

The synthesis strategy for Se-derivative N′-hydroxy-1,2,5-oxadiazole-3-carboximidamide and Composite Sorbents (Se/Si-50) was obtained as a result of the polycondensation reaction of selenium (IV) oxide and N′-hydroxy-1,2,5-oxadiazole-3-carboximidamide, followed by the formation of a nanofilm on the surface of finely dispersed silica gel particles, according to the procedure specified in [32]. The chitosan-based composite sorbent Chit-1/1 was prepared by introducing a chitosan solution with active stirring into a liquid reaction mixture of selenium (IV) oxide and N′-hydroxy-1,2,5-oxadiazole-3-carboximidamide, according to the procedure specified in [33].

### 4.1. Evaluation of Sorption Characteristics Under Static Conditions

The sorption properties of the materials under static conditions were studied by removing uranium from solutions at a volume-to-mass ratio of 1000 mg L^−1^. The experiment consisted of two stages. First, the sorbents were pre-equilibrated in a blank solution (without uranium) for 24 h. The pre-equilibrated sorbents were then separated from this solution and supplemented with the working uranium solution. The initial uranium concentrations ranged from 100 ng·L^−1^ (for ^232^U and ^233^U mixture) to 30 mg L^−1^ (for ^238^U solutions). The mixture was agitated for 24 h. Subsequently, the uranium-containing solution was separated from the sorbent by filtration through a 0.45 µm cellulose acetate membrane filter. Based on data about uranium content in solutions [50], the distribution coefficients (K_d_, mL g^−1^) and recovery efficiency (S, %) were calculated (Equations (1) and (2), respectively)

(1) $$\mathrm{Sorption}=\left(1-\left(\frac{C_{e}}{C_{i}}\right)\right)\times 100,\;\%$$

(2) $$K_{d}=\frac{C_{i}-C_{e}}{C_{i}}\times \frac{V}{m}$$

where C_i_ is the initial concentration of uranium in the model solution (mg L^−1^), C_e_ is the equilibrium residual concentration of uranium in the solution after filtration (mg L^−1^), V is the volume of the solution (mL), and m is the weight of the sorbent sample (g).

The effect of competing anions on the static sorption capacity for uranium was investigated using solutions of EDTA and oxalate (as their sodium salts). To assess the variation in the sorption efficiency of the analyzed adsorbents, the anion concentration was varied from 10^−5^ to 10^−2^ mol L^−1^. Based on these data, the static exchange capacity (SEC, mg g^−1^) was calculated (Equation (3)).

(3) $$SEC=\frac{\left(C_{i}-C_{e}\right)\times V}{m}$$

where C_i_—the initial concentration of uranium in the model solution (mg L^−1^), C_e_—the equilibrium residual concentration of uranium in the solution after filtration (mg L^−1^).

Table 5 demonstrates the compositions of the experimental solutions used for sorption experiments. Seawater was collected in the Sea of Okhotsk in the village of Okhotskoye, Sakhalin Island, Sakhalin Oblast, Russia. River and tap water were collected in Yuzhno-Sakhalinsk city, Sakhalin Island, Sakhalin Oblast, Russia.

The influence of mass transfer on the sorption rate was evaluated using an experimental interruption method, where the sorbent was separated from the model solution and reintroduced after a specified time interval. The U (VI) removal efficiency was calculated using Equation (1).

Kinetic parameters were determined under continuous shaking in a thermostatic shaker. At specific time intervals, solution aliquots were collected, and the residual U (VI) concentration was measured.

The kinetic parameters of uranium adsorption were estimated using the Boyd–Adamson equation [51]. This model allows for the calculation of the effective diffusion coefficient from the diffusion rate constant. The calculation strategy was described in detail in our previous work [33].

### 4.2. Evaluation of Sorption Characteristics Under Dynamic Conditions

The study of the dynamic characteristics of sorption was conducted under continuous flow conditions using a model solution with a pH of 6 or 8 and 0.1 mol L^−1^ NaNO_3_ with uranium concentration 20–30 mg L^−1^. The solution was passed through a fixed sorbent layer with a volume of 1 mL and a grain size of 0.05–0.2 mm at a rate of 10 b.v. h^−1^. Prior to this, the materials were soaked in a model uranium-free solution. Filtrates after the column were divided into fractions, and the uranium content was measured for each fraction. The sorption capacity (*S*u) of the adsorbents was calculated using Equation (4):

(4) $$S_{U}=(C_{i}-C_{e})\times \frac{V}{m}$$

where *C_i_*—initial uranium concentration [mol L^−1^], *C_e_*—the equilibrium uranium concentration [mol L^−1^], *V*—the model solution volume [L], *m*—weight of the sorbent suspension [g].

Based on data adsorption vs. passes solution volume, the dynamic sorption capacity was quantified by the curve integration (Equation (5)).

(5) $$TDEC=\int_{0}^{n}f\left(\frac{\left(C_{0}-C_{i}\right)V_{i}}{m}\right)dV_{t}$$

where *C*_0_, *C*_i_ —content of uranyl ions in the initial model solution and in eluate fraction *i*, [mmol mL^−1^]; *V*_i_—the volume of eluate fraction *i*, [mL]; *m* dry sorbent weight, [g]; *V*_t—_the total solution volume, [mL].

The efficiency of U (VI) desorption was assessed under dynamic conditions at a flow rate of 5 BV h^−1^. First, the adsorbent was saturated with U (VI) in a dynamic mode. Subsequently, a 0.5M NaHCO_3_ and 0.5M NH_4_NO_3_ eluent solution was passed through the column. The eluent was collected in fractions, and the U (VI) content was determined for each. Based on these data, the overall dynamic desorption efficiency was calculated (Equation (6)).

(6) $$Desorption=\left(\frac{\sum_{1}^{i}V_{i}\times C_{i}^{el}}{C_{f}}\right)\times 100$$

where $C_{i}^{el}$—the U (VI) concentration in eluate fraction *i* [mol L^−1^]; $C_{i}^{el}$—the *i* fraction eluate volume [mL]; *C_f_*—the content of U (VI) removed during sorption [mol L^−1^]; *i*—the eluate fraction number.

### 4.3. Equipment

To identify the characteristic features of molecular structure formation in the samples, a set of physicochemical analysis techniques was applied. The types of chemical bonds within the polymer network were identified by infrared spectroscopy. The measurements were carried out using a Spectrum1000 spectrometer (Iraffinity-1S, Shimadzu, Kyoto, Japan), with samples prepared as pellets in KBr.

The phase composition of the synthesized materials and the nature of the interaction between the matrix and the sorption-active component were studied via X-ray diffraction analysis, using a Colibri X-ray diffractometer (JSC IC Burevestnik, Zelenograd, Russian) with CuKα radiation. The data were collected in a point-by-point scanning mode across an angular range of 2° < 2θ < 90°.

An atomic-absorption spectrophotometer AA-7000 (Shimadzu, Kyoto, Japan) was used to analyze the elemental composition of the model samples for studying the materials’ sorption-selective properties.

Uranyl ion concentrations were quantified using a SILab TUV8DCS dual-beam spectrophotometer (Beijing Purkinje General Instrument Co., Ltd., Beijing, China). Absorbance readings were taken across the 600–800 nm wavelength interval. The calibration curve for uranium content had a radionuclide concentration range from 0.1 mg L^−1^ to 30 mg L^−1^.

The thermodynamic parameters of sorption were determined using a water shaker bath STEGLER SB-22 (Stegler, Lanzhou, China) in the temperature range from 30 to 70 °C.

The specific surface area and pore distribution of the studied material were determined by the method of low-temperature adsorption-nitrogen desorption. The measurements were carried out on an Autosorb IQ specific surface area and porosity analyzer (manufactured by Quantachrome Instruments, Boynton Beach, FL, USA). A standard technique based on the Brunauer–Emmett–Teller theory was used to calculate the specific surface area.

## Figures and Tables

**Table 1:** Specific pore volume and specific surface area of the sorbents (via the nitrogen adsorption); kinetic and sorption-selective parameters of uranium adsorption from model solutions of 0.01M NaNO_3_, pH 8.

No.	Parameters	Se-Init	Se/Si-50	Chit-1\1
1	Pore volume, cm^3^/g	0.05	0.51	0.75
2	Specific surface area, cm^3^/g	2	243	420
3	Specific pore size, nm	1.21	12.2	28.0
Kinetic parameters of sorption				
4	t_max_ × 10^−2^ min (continuous)	2.8	2.4	1.2
5	t_max_ × 10^−2^ min (interrupted)	1.6	2.4	0.8
6	K_d_ × 10^−5^, cm^3^/g	0.1	7.9	145.0
7	D_i_ × 10^6^, cm^2^/min	5.3	12.7	28.2
8	T_1/2_, min	32.2	13.3	8.3
9	R^2^	0.992	0.996	0.995
The limiting stage of sorption		Internal diffusion	External diffusion	Internal diffusion

**Table 2:** Calculated parameters of the pseudo-first-order (PFO) and pseudo-second-order (PSO) kinetic models.

Name	Model	Parameter	Temperature, °C		
			30	50	70
Se-am	PFO	*SEC*, mmol g^−1^	0.024 ± 0.001	0.020 ± 0.001	0.020 ± 0.001
		*k*_1_, min^−1^	0.065 ± 0.018	0.128 ± 0.003	0.238 ± 0.021
		*R* ^2^	0.910	0.951	0.950
	PSO	*k*_2_, mmol g^−1^min^−1^	0.875 ± 0.043	4.740 ± 0.340	12.400 ± 3.600
		*R* ^2^	0.972	0.983	0.975
Se/Si-50	PFO	*SEC*, mmol g^−1^	0.032 ± 0.004	0.029 ± 0.004	0.028 ± 0.001
		*k*_1_, min^−1^	0.066 ± 0.008	0.091 ± 0.003	0.199 ± 0.032
		*R* ^2^	0.929	0.926	0.956
	PSO	*k*_2_, mmol g^−1^min^−1^	5.310 ± 0.460	8.500 ± 0.250	14.800 ± 2.600
		*R* ^2^	0.967	0.964	0.973
Chit-1\1	PFO	*SEC*, mmol g^−1^	0.034 ± 0.002	0.031 ± 0.001	0.023 ± 0.002
		*k*_1_, min^−1^	0.015 ± 0.003	0.049 ± 0.001	0.097 ± 0.033
		*R* ^2^	0.932	0.895	0.925
	PSO	*k*_2_, mmol g^−1^min^−1^	9.770 ± 0.230	12.630 ± 0.120	8.700 ± 1.940
		*R* ^2^	0.992	0.987	0.989

**Table 3:** Comparative sorption characteristics of adsorbents for uranium extraction under dynamic conditions.

Sorbent Type	Sorbent Mass, g	Solution	Pumping Speed, L min^−1^	C (U), mg L^−1^	TDEC, mg g^−1^	Effective Filtration Cycle	Reference
PAO/PVA hydrogel nanofibers	0.01	SWS	1.5	8	13.4	-	[39]
BUT-12-3COOH (MOΦ)	0.2	H_2_O distilled	3.0	10	-	300 b.v. (100%)	[40]
Phosphate-based hypercrosslinked polymers (HCPs)	0.2	H_2_O distilled, pH 7	1.0	50	-	-	[11]
AOP@ZIF-8/TA и AOP MOC	0.01	SW	5.0	10	11.2	-	[41]
Polyamine-grafted composite resin	0.9427	GWS, pH 7	1.0	1	12.5	1000 b.v. *	[42]
	0.9427	TW, pH 7.3	1.0	1	-	1500 b.v. *	[42]
PMOF-3	1	H_2_O distilled, pH 6	1.0	11.9	11.4	2500 b.v. *	[18]
Silk-like fiber based on super uranyl-binding protein (SUP)	0.01	SW	160.0	16	13.7	-	[43]
SAP composites (SA/PAO/PEI)	0.05	SW	25.0	10	20.3	-	[44]
Se/Si-50	0.7	SW	1	30	15.5	1045 b.v. *	This study
Chit-1\1	0.7	SW	1	30	25.3	2960 b.v. *	This study

*—the volume of the purified filtrate, before reducing the values of adsorption efficiency to 50%; SW—seawater; SWS—seawater simulation; GWS—groundwater simulation; TW—tap water.

**Table 4:** Parameters of the molecular lattice before and after uranium sorption.

Sample Description	State	Diffraction Angle, 2θ, deg.	Interplanar Spacing (d), Å	CSR Size (L), nm
Se-am	Before sorption	27.36	0.8585	12.08 ± 2.35
	After sorption	27.27	0.8797	10.94 ± 2.14
Se/Si-50	Before sorption	27.24	0.8862	7.01 ± 2.02
	After sorption	27.28	0.8472	9.12 ± 4.62
Chit-1/1	Before sorption	29.27	0.8767	2.70 ± 0.09
	After sorption	29.61	0.9811	0.36 ± 0.03

**Table 5:** Composition of the experimental solutions.

Ion	Component Content, mg/L		
	TW	RW	SW
Na^+^	–	24.1	10,500
K^+^	–	2.1	380
Mg^2+^	1.9	6.1	1350
Ca^2+^	5.8	5.0	400
Fe^2+^	0.4	–	0.01
Cl^−^	3.8	32.5	19,000
SO_4_^2−^	14.5	14.5	4.66
CO_3_^2−^	–	42.7	48
Br^−^	–	–	0.07
F^−^	–	–	1.3
Si	4.3	5.9	3
pH	6.5–7.2	6.9–7.5	7.7–8.4

## Data Availability

The data presented in this study are available on request from the corresponding author. The data are not publicly available due to ongoing researches using a part of the data.
